# Supplementary material for: Addressing missing data in randomized clinical trials: A causal inference perspective
Source: PLoS One. 2020 Jul 6;15(7):e0234349. doi: 10.1371/journal.pone.0234349 (PMC7337281; doi:10.1371/journal.pone.0234349)
Supplement: S1 Appendix — (DOCX) [file pone.0234349.s001.docx]

**Appendix A**

In Appendix A, we derive Equation 3. We define a full sample (*fs*) that includes all observed and missing outcome, and an observed sample (*os*). For expositional reasons, but without loss of generalization, assume that $\beta$ can only be expected to be positive. Then, to find the required sample size such that exactly 80% of the average treatment effect estimates will be statistically significantly different from 0, the following should hold:

| $0+1.96\cdot{SE}_{fs}=\beta-0.84\cdot{SE}_{fs}$. | (7) |
| --- | --- |

The left-hand side of this equation indicates that the treatment effect should be at least 1.96 standard errors away from 0 in order to attain statistical significance. The right-hand side reflects that the treatment effect $\beta$ should be an additional $0.84$ standard errors larger, as for the corresponding randomized clinical trial to have an associated statistical power of 80% [19]. Without loss of generality, we conveniently assume equal group sizes and that the variation is the same in both groups ($\sigma_{treatment}=\sigma_{control}=\sigma_{fs}$), such that ${SE}_{fs}=\frac{2\sigma_{fs}}{\sqrt{N_{fs}}}$ and we can solve for the required sample size conditional on the expected value for $\beta$ [19].

The following two equations have to be considered to link power reduction to missing outcome observations and a corresponding smaller observed sample size $N_{os}$:

| $\beta_{fs}=$ $1.96\cdot{SE}_{fs}$+$0.84\cdot{SE}_{fs}=$ $2.8\cdot{SE}_{fs}$ | (8) |
| --- | --- |
| $\beta_{os}=$ $1.96\cdot{SE}_{os}+ \theta\cdot{SE}_{os}$. | (9) |

Equation 8 is the rearranged version of equation 7, such that $\beta_{fs}$ is isolated on the left-hand side. Under the assumption that $N_{fs}$ was chosen such that the power of the randomized clinical trial was set at exactly 80%, Equation 7 represents the implications for observing ${N_{os}<N}_{fs}$ in which $\theta$ reflects that the trial has less than 80% power due to the reduced sample size (i.e. $\theta<0.84$).

An important feature is that both $\beta_{fs}$ and $\beta_{os}$ are unbiased estimators of the treatment effect if and only if missing outcome observations are truly random events. By acknowledging that $\beta_{fs}=\beta_{os}$ and $\sigma_{fs}=\sigma_{os}$, Equation 9 can be substituted in Equation 8, after which solving for $\theta$ leaves:

| $\theta=2.8\cdot\frac{{SE}_{fs}}{{SE}_{os}}-1.96$  = $2.8\cdot\sqrt{\frac{N_{os}}{N_{fs}}}-1.96$ | (10) |
| --- | --- |

Equation 10 shows the power reduction as a result of sample size reduction. A standard normal table, which present the values of the cumulative distribution function of the normal distribution, indicates that $\theta$=0.84 is corresponding to a power of 80%. Yet, when $\sqrt{\frac{N_{os}}{N_{fs}}}$ is, for example, .75 generates a value for $\theta$ of 0.14, yielding a power of 56 percent and –thus- a power reduction of 24 percentage points.

19. Gelman, A. & Hill, J. (2006) Data Analysis using Regression and Multlevel/Hierarchical Models, Cambridge University Press, New York.
