# Supplementary material for: Addressing missing data in randomized clinical trials: A causal inference perspective
Source: PLoS One. 2020 Jul 6;15(7):e0234349. doi: 10.1371/journal.pone.0234349 (PMC7337281; doi:10.1371/journal.pone.0234349)

**Appendix B**

This appendix provides a detailed outline of simulated data attrition for demonstrating the RFLB approach. First, the experimental sample was expanded by 10, such that the number of observations is larger (N=1930) and power issues are no longer a concern, while leaving the average treatment effect unaffected. The simulated attrition variable was constructed, such that it was jointly associated with treatment, pre-score and gender. It is important to recognized that conditioning on background characteristics can only produce tighter bounds if there exists a statistical dependency between attrition, treatment and, respectively, pre-scores and gender.

Attrition was simulated by conducting the following 3 steps. In step one, a random uniform (0,1) variate *R* was generated]. In step 2, pre-score were ranked -from low to high- and a categorical pre-score variable (*PreC)* was generated breaking down pre-scores into five evenly spaced categories. In step three, a binary attrition variable was simulated using the following 13 rules:

1. Attrition = 1 if *R ≤* 0.05 & *T* = 0
2. Attrition = 1 if *R ≤* 0.15 & *T* = 1
3. Attrition = 1 if *R ≤* 0.10 & *T* = 0 & *PreC* = 2
4. Attrition = 1 if *R ≤* 0.15 & *T* = 0 & *PreC* = 3
5. Attrition = 1 if *R ≤* 0.25 & *T* = 0 & *PreC* = 4
6. Attrition = 1 if *R ≤* 0.45 & *T* = 0 & *PreC* = 5
7. Attrition = 1 if *R ≤* 0.10 & *T* = 1 & *PreC* = 1
8. Attrition = 1 if *R ≤* 0.20 & *T* = 1 & *PreC* = 2
9. Attrition = 1 if *R ≤* 0.40 & *T* = 1 & *PreC* = 3
10. Attrition = 1 if *R ≤* 0.60 & *T* = 1 & *PreC* = 4
11. Attrition = 1 if *R ≤* 0.80 & *T* = 1 & *PreC* = 5
12. Attrition = 1 if *R ≤* 0.25 & *T* = 0 & *Female* = 0
13. Attrition = 1 if *R ≤* 0.40 & *T* = 1 & *Female* = 0

Rules 1 and 2 ensure that there is an attrition difference between the control and treatment group of 10 percent. Rules 3 up to 11 ensure (1) that attrition is an increasing function of pre- scores, and (2) that this relationship is stronger for participants in the treatment group. Rules 12 and 13 ensure that there is more attrition for men than for women and, -again- that attrition is even higher for men in the treatment group.

Figure 6 shows the increasing relationship between the simulated attrition and pre- scores. The mean simulated attrition rate was .36.

**Figure 6:** Simulated attrition by pre-scores


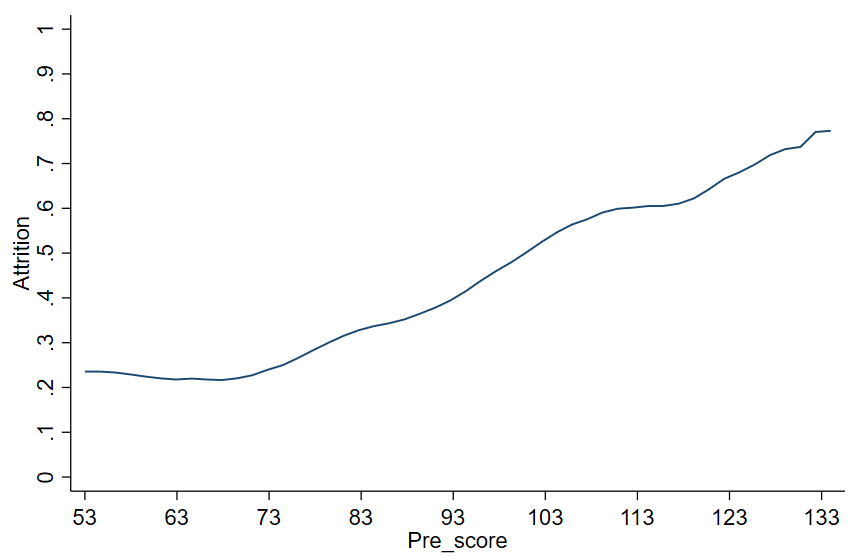


Figure 7 shows the resulting interdependency between treatment and, respectively, pre-scores and gender in relation to the simulated attrition variable after applying Rule 1-13.

**Figure 7:** Simulated attrition and pre-scores by gender and treatment status


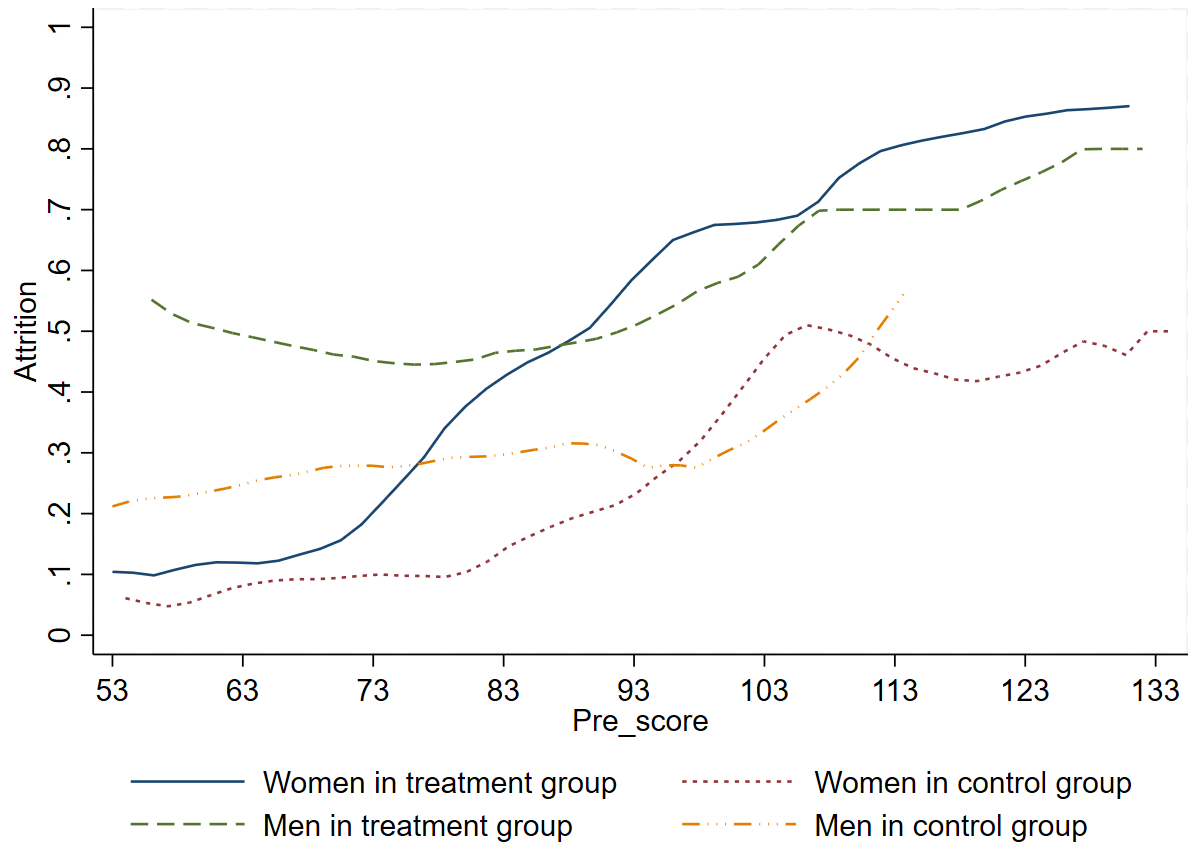

Supplement: S2 Appendix — (DOCX) [file pone.0234349.s002.docx]
