# Supplementary material for: Addressing missing data in randomized clinical trials: A causal inference perspective
Source: PLoS One. 2020 Jul 6;15(7):e0234349. doi: 10.1371/journal.pone.0234349 (PMC7337281; doi:10.1371/journal.pone.0234349)
Supplement: Attachment — Submitted filename: Reply to Reviewer 1.docx [file pone.0234349.s005.docx]

Reply to Referee 1

For: PLOS ONE

Manuscript ID: PONE-D-20-02997

Manuscript Title: Addressing Missing Data in Randomized Clinical Trials: A Causal Inference Perspective

Dear Referee,

We have received your comments on our submitted paper to PLOS ONE*.* First, let us express our gratitude for your constructive remarks and suggestions. We feel that we were able to meet most of your concerns.

Below, we address each of your comments in detail.

Reviewer #1:

- *The paper is well-written but could be improved further with more relevance to readers with the inclusion of (1) simulations with different baseline covariate distributions under different treatment effect sizes: small, medium and large.*

**Reply:**

Thank you for your kind words and for this great advice. We have now performed the bounding approach using effect sizes $d=0.2$, $d=0.4$ and $d=0.8$ to show how the use of random forest lee bounds tighten the unconditional lee bounds. We feel that the paper has improved greatly by the inclusion of these simulations. The text now reads as follows:

To generalize the bounding estimation results we also performed simulations in which the bounding estimates were again obtained under different treatment sizes: small, medium and large (i.e. $d=0.2$, $d=0.4$ and $d=0.8$). These bounding results are shown in Table 2 and the results suggest that the unconditional Lee bounds are tightened by the RFLB approach with 19.8, 16.8 and 16.6 percent for respectively a small, medium and large treatment size. This is an important result: when the effect size becomes smaller it is more likely that 0 lies and thus more important to obtain tighter bounds (page 21, starting with the last paragraph).

**Table 2:** Bounding Estimation Results for Small, Medium and Large Effect Sizes

|  |  |  |  |  | **95% conf. interval** | | |  | |  |  |
| --- | --- | --- | --- | --- | --- | --- | --- | --- | --- | --- | --- |
|  | **Lower** | **Upper** | **Diff.** | **%-gain** | **Lower** | **Upper** | **Diff.** | | **%-gain** | |  |
| **Unconditional Lee Bound** |  |  |  |  |  |  |  | |  | |  |
| Small (d=0.2) | -0.280 | -0.162 | 0.118 | . | -0.297 | -0.145 | 0.153 | | . | |  |
| Medium (d=0.4) | -0.560 | -0.325 | 0.235 | . | -0.595 | -0.289 | 0.306 | | . | |  |
| Large (d=0.8) | -1.119 | -0.650 | 0.470 | . | -1.189 | -0.578 | 0.610 | | . | |  |
|  |  |  |  |  |  |  |  | |  | |  |
| **Conditional Lee Bound** (Female) |  |  |  |  |  |  |  | |  | |  |
| Small (d=0.2) | -0.281 | -0.164 | 0.117 | 0.825 | -0.298 | -0.147 | 0.152 | | 0.655 | | |
| Medium (d=0.4) | -0.557 | -0.326 | 0.231 | 1.836 | -0.591 | -0.291 | 0.300 | | 1.768 | | |
| Large (d=0.8) | -1.123 | -0.644 | 0.479 | -1.961 | -1.193 | -0.573 | 0.620 | | -1.540 | | |
|  |  |  |  |  |  |  |  | |  | | |
| **Random Forest Lee Bound** (Female, Pre-score) |  |  |  |  |  |  |  | |  | | |
| Small (d=0.2) | -0.269 | -0.175 | 0.094 | 19.818 | -0.287 | -0.155 | 0.132 | | 13.294 | | |
| Medium (d=0.4) | -0.533 | -0.338 | 0.196 | 16.802 | -0.570 | -0.297 | 0.272 | | 10.933 | | |
| Large (d=0.8) | -1.069 | -0.677 | 0.392 | 16.609 | -1.142 | -0.597 | 0.545 | | 10.665 | | |

- *and (2) relevant data and syntax to enable replication of authors findings.*

The simulation data and STATA-code is now available. We have uploaded the STATA data and do-file to the journal such that users can conduct the bounds analysis. We would like to emphasize that our plan for the future is to write a STATA routine such that everyone has access to a STATA ADO-file that can be used to directly apply the unconditional, the conditional and the RFLB method.
